# Osteoprotegerin as a Marker of Atherosclerosis in Diabetic Patients

**DOI:** 10.1155/2013/182060

**Published:** 2013-01-17

**Authors:** Areti Augoulea, Nikolaos Vrachnis, Irene Lambrinoudaki, Konstantinos Dafopoulos, Zoe Iliodromiti, Angelos Daniilidis, Michail Varras, Andreas Alexandrou, Efthymios Deligeoroglou, George Creatsas

**Affiliations:** ^1^2nd Department of Obstetrics and Gynecology, University of Athens Medical School, Aretaeio Hospital, 11526 Athens, Greece; ^2^Obstetric-Gynecological Unit and Research Center, Evgenideio Hospital, University of Athens, 11526 Athens, Greece; ^3^Department of Obstetrics and Gynaecology, Medical School, University of Thessaly, 41334 Larissa, Greece; ^4^2nd University Department of Obstetrics and Gynecology, Hippokratio General Hospital, University of Thessaloniki Medical School, 54642 Thessaloniki, Greece; ^5^Department of Obstetrics and Gynaecology, General District Hospital “Helena Venizelou”, 11521 Athens, Greece; ^6^1st Department of Surgery, University of Athens Medical School, Laiko Hospital, 11527 Athens, Greece

## Abstract

Atherosclerosis is the principal cause of cardiovascular disease (CVD) and has many risk factors, among which is diabetes. Osteoprotegerin (OPG) is a soluble glycoprotein, involved in bone metabolism. OPG is also found in other tissues, and studies have shown that it is expressed in vascular smooth muscle cells. OPG has been implicated in various inflammations and also has been linked to diabetes mellitus. Increased serum OPG levels were found in patients with diabetes and poor glycemic control. Furthermore, prepubertal children with type 1 diabetes have significantly increased OPG levels. Receptor activator of nuclear factor kappa-B ligand (RANKL) is not found in the vasculature in normal conditions, but may appear in calcifying areas. OPG and RANKL are important regulators of mineral metabolism in both bone and vascular tissues. Few data are available on the relationship between plasma OPG/RANKL levels and endothelial dysfunction as assessed using noninvasive methods like ultrasound indexes, neither in the general population nor, more specifically, in diabetic patients. The aim of our review study was to investigate, based on the existing data, these interrelationships in order to identify a means of predicting, via noninvasive methods, later development of endothelial dysfunction and vascular complications in diabetic patients.

## 1. Introduction

Atherosclerosis is the principal cause of cardiovascular disease (CVD) and has numerous risk factors, among which diabetes. Over recent years, it has been determined that atherosclerosis is the result of a systemic inflammatory process involving immune and vascular cells. Osteoprotegerin (OPG) is a soluble glycoprotein, mainly involved in bone metabolism, but it is also found in various other tissues like vascular smooth muscle cells. OPG has been implicated in various inflammations and has additionally been linked to diabetes mellitus, silent myocardial ischemia, acute myocardial infarction, and left ventricular dysfunction [1].

Patients with type 1 diabetes mellitus seem to be at risk of low bone mass [2] and osteoporosis [3, 4]. These patients also have accelerated atherosclerosis with vascular calcification, which is associated with increased morbidity and mortality due to vascular disease [5]. Patients with diabetes and poor glycemic control were found to have increased OPG levels. Increased plasma OPG concentrations are associated with coronary artery disease [6, 7] frequently accompanied by atherosclerosis, stroke, and vascular mortality [8, 9] as well as by subclinical atherosclerosis [10, 11] and overall cardiovascular morbidity and mortality [12] in obese nondiabetic subjects [13]. Among type 2 diabetes patients, a strong correlation of OPG levels and angiopathy was established [14, 15]. Furthermore, prepubertal children with type 1 diabetes have significantly increased OPG levels [16]. High-resolution ultrasound is a reliable noninvasive method for detecting early structural and functional atherosclerotic changes in the arterial wall. Carotid intima-media thickness (CIMT) is a structural marker of early atherosclerosis that correlates with vascular risk factors. It correlates with the severity and the extent of coronary artery disease predicting the likelihood of cardiovascular events [17, 18]. CIMT is a predictor of vascular events in the future [19]. Patients with type 2 diabetes and impaired glucose tolerance have increased CIMT [20]. Flow-mediated dilatation (FMD) of the brachial artery is an ultrasound marker of endothelial function. Two studies found both increased IMT and impaired FMD in young children with risk factors for atherosclerosis, such as diabetes [20–22]. However, few data are available on the relationship between plasma OPG and RANKL and diabetic patients alone and between these levels and endothelial dysfunction assessed with ultrasound indexes of subclinical atherosclerosis like CIMT. The aim of our paper was to investigate these relationships according to the existing data in the recent literature.

## 2. OPG/RANKL

OPG is a secreted member of the tumor necrosis factor (TNF) receptor superfamily which was initially found in bone [23]. It functions as a strong anti-resorptive factor and exerts its effect through binding and neutralizing the receptor activator for NF-kB ligand (RANKL). RANKL is a cytokine with strong osteoclast-inducing activity [24].

OPG is also found in other mesenchymal tissues, and in vitro studies have shown that OPG is expressed in vascular smooth muscle cells [25, 26] and acts as a survival factor for the endothelial cells [27]. The tissue concentration of OPG in aorta and hip-bone is almost identical but 500 times higher than the plasma concentration [28]. OPG and RANKL are important regulators of mineral metabolism in both bone and vascular tissues [29]. The function of OPG in the arterial wall is not known, but it is hypothesized to play an important role in the vasculature. Both experimental [30] and human studies [31] have shown that OPG is present in the arterial wall [32]. In bone OPG inhibits bone resorption, whereas RANKL promotes bone resorption in contrast to their action in the vasculature where RANKL promotes calcification and OPG has a protective effect [29]. Denosumab, a fully human monoclonal antibody to the receptor activator of nuclear factor-*κ*B ligand (RANKL), mimics OPG in its ability to bind and neutralize RANKL. Cummings et al. after a 3-year treatment of osteoporotic postmenopausal women with denosumab found no association with cardiovascular disease [33]. There are experimental data supporting this theory. Bucay et al. studied the physiological role of OPG in OPG-deficient mice. These adolescent and adult mice had a high incidence of fractures and also exhibited medial calcification of the aorta and renal arteries. The conclusion of the study was that OPG knock-out mice develop arterial calcifications; thus this molecule may act as a vascular calcification inhibitor [34]. Furthermore, Bennett et al. assessed whether OPG plays a role in the progression and calcification of advanced atherosclerotic lesions in mice deficient in both OPG and apolipoprotein E (OPG^−/−^, Apo E^−/−^ mice), and the result was that OPG inhibits advanced plaque progression by preventing increase in lesion size and lesion calcification [35]. It has also been shown by Price et al. that doses of recombinant OPG that inhibit bone resorption are able to potently inhibit the calcifications of arteries induced by warfarin or vitamin D treatment of rats [36].

Accordingly, overall it seems that OPG is an important regulating molecule in bone turnover, while plasma OPG has been shown to correlate to bone and arterial diseases [37]. Available data so far cannot demonstrate whether there is a causal relationship between increasing OPG and CVD or elevated OPG is a compensatory epiphenomenon of developing arterial lesions.

## 3. OPG as a Marker of Atherosclerosis in the General Population

 OPG, which has been shown to be present in atherosclerotic plaques [8], might be involved in vascular calcification [38]. Data from the Dallas Heart Study have shown that OPG is associated with coronary artery calcium and aortic plaque in an unselected population [8]. OPG has moreover been shown to enhance the matrix content in plaques [30]. Data suggest that OPG is induced by atherosclerosis and may be upregulated as an incomplete compensatory response to the vessel insult, possibly thereby limiting vascular calcification [31].

High plasma concentrations of OPG have been associated with coronary artery atherosclerosis [32, 39]. In a large population study it was observed that OPG appears to be a promising biomarker of atherosclerosis [40]. Browner et al. reported that serum OPG levels were associated with cardiovascular mortality in a large cohort of postmenopausal women [37]. A recent study suggested that a higher level of OPG is an independent predictive factor of prognosis in patients with intermediate coronary lesions [41]. OPG has been used to predict long-term mortality in patients with acute coronary syndrome [42]. Furthermore, it was found to predict mortality in patients with ischemic stroke [43]. In a recent study, Pedersen et al. assessed serum OPG as a predictor of long-term prognosis in patients with suspected stable angina pectoris undergoing elective coronary angiography. In patients with stable angina pectoris, elevated serum OPG levels were associated with increased risk of all-cause mortality, cardiovascular disease mortality, and myocardial infarction [44].

Previous studies have reported various associations between serum OPG levels and incidence of ischemic strokes and cerebrovascular deaths [12, 37, 45], whereas most prospective studies have shown that OPG can predict cardiovascular events and mortality [9, 37, 39, 45, 46]. Semb et al. examined the association between serum levels of OPG and RANKL with future coronary artery disease in apparently healthy individuals. OPG levels were found to correlate well with the risk of future CAD in apparently healthy subjects, independently of other cardiovascular risk factors [47]. In a very recent study, Zagura et al. evaluated the association between OPG and arterial stiffness both for patients with peripheral arterial disease and for healthy controls and found an independent association between OPG and radial and aortic pulse wave velocity in patients with peripheral arterial disease and in controls alike. The suggestion was made that the calcification inhibitor OPG may worsen vascular stiffness in atherosclerotic as well as in clinically healthy subjects [47].

## 4. OPG as a Marker of Atherosclerosis in Diabetic Patients

### 4.1. OPG and CVD Events

The pathophysiological connection between plasma OPG concentrations and CVD is not known, but a correlation with both arterial and myocardiac disease has been suggested [48, 49]. Kiechl et al. showed a strong association between serum OPG levels and cardiovascular risk factors, including diabetes [9]. 

Clinical studies suggest that serum OPG levels increase in association with vascular calcification, coronary artery disease, and stroke. Of note, plasma OPG levels have been demonstrated as being an independent risk factor for the 10-year incidence of CVD and vascular mortality [9]. Two cross-sectional studies of subjects undergoing coronary angiography revealed a strong positive association between OPG serum levels and advanced CAD [6, 7]. Ueland et al. reported on the efficacy of OPG as a novel marker of cardiovascular mortality and clinical events in patients with acute myocardial infarction [49]. Crisafulli et al. studied serum OPG and RANKL levels in patients with ST elevation in acute myocardial infarction and found increased serum OPG levels [50]. 

 Kiechl et al. showed a strong association between serum OPG levels and cardiovascular risk factors, including diabetes [9]. Avignon et al. investigated OPG concentrations and signs of myocardial ischemia perfusion on myocardial perfusion scintigraphy in patients with type 1 or 2 diabetes and concluded that OPG measurement can help to better define the diabetic population with an increased risk of developing SMI [51]. Rasmussen et al. suggested that OPG levels were associated with the rate of glycemic control and CVD risk in patients with type 1 diabetes [52] (Table 1). A very recent study in type 2 diabetic patients observed the relationship between serum OPG and vascular alterations. The study group assessed the relationship of OPG serum levels with basal glycemia, glycosylated hemoglobin, blood pressure, and endothelial dysfunction and suggested that OPG is an indicator of the level of control of diabetes, endothelial dysfunction, and cardiovascular risk [53].

### 4.2. OPG and Subclinical Atherosclerosis

There is increasing evidence that endothelial dysfunction and increased CIMT are precursors of clinically detectable atherosclerosis [54]. Diabetes mellitus is an established risk factor for early development of accelerated atherosclerosis and microangiopathy [55]. Vascular complications of diabetes are not clinically evident in diabetic children. However, subclinical vascular involvement in the form of impaired endothelial function and increased CIMT has been demonstrated even in young subjects with diabetes mellitus [56].

Markers of micro- and macrovascular disease are needed in diabetes in order to identify patients at risk of severe complications. Olesen et al. observed significantly higher concentrations of OPG in arteries from patients with diabetes compared to nondiabetics [28]. Another observational study found higher plasma OPG concentrations in diabetic individuals compared with nondiabetics, although the absolute concentration difference was limited [37]. The epicoronary artery stenosis which is caused by atherosclerosis is shown to correlate well with plasma OPG levels in type 2 diabetes [23]. A recent study investigated 166 type 2 diabetic patients and found that plasma OPG was associated with age, glycemic control, and microalbuminuria [57]. Grauslund et al. investigated in a cohort study OPG as a noninvasive marker of micro- and macrovascular complications in long-term type 1 diabetic patients and found some associations of OPG with nephropathy [58]. 

Although it has been demonstrated that increased CIMT and the presence of carotid plaques are correlated with plasma OPG levels in healthy individuals [9, 59], only recently have relevant data been published concerning type 2 diabetic patients [60]. In a very recent study, increased plasma OPG concentration was associated with carotid and peripheral arterial disease in patients with type 2 diabetes. These type 2 diabetes patients without known CVD were subsequently referred to a diabetes clinic for the first time and were screened for carotid arterial disease, peripheral arterial disease, and myocardial ischemia [61]. Ishiyama et al. found that CIMT was positively correlated to plasma OPG levels in patients with type 2 diabetes [62]. Shin et al. demonstrated that elevated OPG levels were significantly associated with endothelial dysfunction in type 2 diabetes [63]. OPG may thus act as an important regulator in the development of vascular dysfunction in diabetes [64]. 

Women with pGDM are well known to have a risk of between 18% and 50% for developing type 2 diabetes mellitus within 5 years following pregnancy, and diabetes is an established risk factor for CVD. In addition, women with a history of GDM are at increased risk of other cardiovascular risk factors, such as obesity, hypertension, dyslipidemia, and subclinical atherosclerosis [65]. Some studies have reported increased CIMT in women with previous gestational diabetes mellitus (pGDM) [66, 67]. Tarim et al. demonstrated increased CIMT in a Turkish cohort including women with pGDM [68] (Table 1). 

Many studies demonstrated impaired endothelium-dependent arterial dilatation in type 1 diabetes patients [23]. In order to determine the presence of subclinical atherosclerosis in type 1 diabetic subjects, Abdelghaffar et al. conducted a study in which they found that the mean CIMT was higher in the adolescents with type 1 diabetes by comparison with the controls [69]. Xiang et al. in their study investigated the relationship between plasma OPG levels and endothelium-dependent arterial dilatation in type 1 diabetic patients and showed that plasma OPG levels were elevated in newly diagnosed type 1 diabetic patients and that plasma OPG levels were significantly associated with endothelial function [70]. Finally, Singh et al. suggested that endothelial function is impaired in children with diabetes mellitus within the first decade of its onset and precedes an increase in CIMT [20], while another study found that impaired FMD response is a common manifestation in children with type 1 diabetes and that it is associated with increased carotid artery thickness, suggesting that endothelial dysfunction in children with type 1 diabetes may predispose them to the development of early atherosclerosis [21]. 

## 5. Conclusions

As cardiovascular morbidity is high in diabetic patients, it is evidently crucial to establish noninvasive methods for monitoring vascular changes such as CIMT and biochemical markers of increased risk for CVD events such as OPG/RANKL. These markers could be used in the clinical setting for the early diagnosis of subclinical atherosclerosis, which would allow for strategies to be designed to reduce the cardiovascular event rate in those patients. 

However, unfortunately the existing data are as yet sparse. Thus, further prospective studies are needed to establish whether increased OPG/RANKL levels and/or CIMT in diabetic patients can in fact predict later development of endothelial dysfunction and vascular complications. 

## Figures and Tables

**Table 1:** Studies evaluating the association of OPG levels with indices of glycemic control, subclinical atherosclerosis, or cardiovascular events in diabetic patients.

Study	Population	Results
Olesen et al., 2005 [28]	21 type 1/type 2 patients	Increased OPG levels in the aortic tunica media are associated with vascular calcifications
Browner et al., 2001 [37]	35 type 2 patients	OPG is associated with cardiovascular mortality
Avignon et al., 2007 [51]	465 type 1/type 2 patients	OPG is associated with silent myocardial ischemia
Rasmussen et al., 2006 [52]	400 type 1 patients	OPG is associated with poor glycemic control and CVD
Blazquez-Medela et al., 2012 [53]	52 type 2 patients	OPG is an indicator of endothelial dysfunction and CVD risk
Altinova et al., 2011 [57]	166 type 2 patients	OPG is associated with poor glycemic control and microalbuminuria
Grauslund et al., 2010 [58]	200 type 1 patients	OPG is associated with nephropathy
Poulsen et al., 2011 [61]	735 type 2 patients	OPG is associated with carotid and peripheral arterial disease
Ishiyama et al., 2009 [62]	168 type 2 patients	OPG is positively associated with vascular calcifications and CIMT
Shin et al., 2006 [63]	104 type 2 patients	OPG levels are associated with endothelial dysfunction
Terekeci et al., 2009 [64]	42 type 2 patients	OPG levels are associated with neuropathy
Xiang et al., 2007 [70]	22 type 1 patients	OPG are associated with endothelial function
